# Central nervous system immune-related disorders after SARS-CoV-2 vaccination: a multicenter study

**DOI:** 10.3389/fimmu.2024.1344184

**Published:** 2024-02-05

**Authors:** Alberto Vogrig, Sara Tartaglia, Marta Dentoni, Martina Fabris, Francesco Bax, Marco Belluzzo, Lorenzo Verriello, Daniele Bagatto, Matteo Gastaldi, Pierluigi Tocco, Marco Zoccarato, Luigi Zuliani, Andrea Pilotto, Alessandro Padovani, Macarena Villagrán-García, Vincent Davy, Gian Luigi Gigli, Jérôme Honnorat, Mariarosaria Valente

**Affiliations:** ^1^ Department of Medicine (DMED), University of Udine, Udine, Italy; ^2^ Clinical Neurology, Department of Head-Neck and Neuroscience, Azienda Sanitaria Universitaria Friuli Centrale (ASU FC), Udine, Italy; ^3^ Institute of Clinical Pathology, Department of Laboratory Medicine, Azienda Sanitaria Universitaria Friuli Centrale (ASU FC), Udine, Italy; ^4^ Neurology Unit, Department of Head-Neck and Neuroscience, Azienda Sanitaria Universitaria Friuli Centrale (ASU FC), Udine, Italy; ^5^ Department of Diagnostic Imaging, Unit of Neuroradiology, Azienda Sanitaria Universitaria Friuli Centrale (ASU FC), Udine, Italy; ^6^ Neuroimmunology Laboratory, IRCCS Mondino Foundation, Pavia, Italy; ^7^ Neurology and Stroke Unit, “Spirito Santo” Hospital of Pescara, Pescara, Italy; ^8^ UOC Neurologia O.S.A. - Azienda Ospedale Università di Padova, Padua, Italy; ^9^ Neurology Unit, AULSS8 Berica, San Bortolo Hospital, Vicenza, Italy; ^10^ Neurology Unit, Department of Clinical and Experimental Sciences, University of Brescia, Brescia, Italy; ^11^ Neurology Unit, Department of Continuity of Care and Frailty, ASST Spedali Civili Brescia University Hospital, Brescia, Italy; ^12^ Laboratory of Digital Neurology and Biosensors, University of Brescia, Brescia, Italy; ^13^ French Reference Centre for Paraneoplastic Neurological Syndromes and Autoimmune Encephalitis, Hospices Civils de Lyon, Hôpital Neurologique, Bron, France; ^14^ MeLiS - UCBL-CNRS UMR 5284 - INSERM U1314, Université Claude Bernard Lyon 1, Lyon, France; ^15^ Department of Neurology, Hôpital Pitié Salpétrière, Assistance Publique des Hôpitaux de Paris, Paris, France

**Keywords:** neurologic adverse events, vaccination, neurological complications, vaccine, COVID-19, SARS-CoV-2

## Abstract

**Background:**

COVID-19 vaccines have been approved due to their excellent safety and efficacy data and their use has also permitted to reduce neurological complications of SARS-CoV-2. However, clinical trials were underpowered to detect rare adverse events. Herein, the aim was to characterize the clinical spectrum and immunological features of central nervous system (CNS) immune-related events following SARS-CoV-2 vaccination.

**Methods:**

Multicenter, retrospective, cohort study (December 1, 2020-April 30, 2022). Inclusion criteria were (1) *de novo* CNS disorders developing after SARS-CoV-2 vaccination (probable causal relationship as per 2021 Butler criteria) (2); evidence for an immune-mediated etiology, as per (i) 2016 Graus criteria for autoimmune encephalitis (AE); (ii) 2015 Wingerchuk criteria for neuromyelitis optica spectrum disorders; (iii) criteria for myelitis.

**Results:**

Nineteen patients were included from 7 tertiary referral hospitals across Italy and France (one of them being a national referral center for AE), over almost 1 year and half of vaccination campaign. Vaccines administered were mRNA-based (63%) and adenovirus-vectored (37%). The median time between vaccination and symptoms onset was 14 days (range: 2-41 days). CSF was inflammatory in 74%; autoantibodies were detected in 5%. CSF cytokine analysis (n=3) revealed increased CXCL-10 (IP-10), suggesting robust T-cell activation. The patients had AE (58%), myelitis (21%), acute disseminated encephalomyelitis (ADEM) (16%), and brainstem encephalitis (5%). All patients but 2 received immunomodulatory treatment. At last follow-up (median 130 days; range: 32-540), only one patient (5%) had a mRS>2.

**Conclusion:**

CNS adverse events of COVID-19 vaccination appear to be very rare even at reference centers and consist mostly of antibody-negative AE, myelitis, and ADEM developing approximately 2 weeks after vaccination. Most patients improve following immunomodulatory treatment.

## 1 Introduction

Prevention of infectious diseases through immunization is one of the greatest public health accomplishments of all time. Like all therapies, vaccination also carries the risk of adverse events. However, the risk of serious events was documented to be very low for all approved types of vaccination and neurological adverse events are exceedingly rare (1).

Notable examples include the 1976 H1N1 influenza vaccine which was withdrawn from the market in the USA due to a small increase of Guillain Barré syndrome (GBS) cases (an additional one case of GBS for every 100,000 vaccines (2)), or the intranasal influenza vaccine withdrawn in Switzerland in 2001 due to a small increase in peripheral facial palsy (an extra 13 cases of Bell’s palsy per 10,000 vaccinations (3)).

However, all cases of reported neurological complications following vaccination should be carefully evaluated to confirm if a neurological disorder is indeed present (which is not obvious as a variety of functional neurological disorders were reported after vaccination (4)) as well as to prove causality (1, 5). The latter aspect is difficult to accomplish as there are no distinguishing clinicopathologic findings in vaccine-induced neurological adverse events (1). Nevertheless, as vaccine uptake is directly influenced by public confidence in the safety of the vaccine, it is crucial that those reporting potential neurological adverse events employ rigorous methodological strategies when determining causal relationships (5).

Coronavirus disease 19 (COVID-19) vaccines comprised adenovirus-vectored preparations, including ChAdOx1nCoV-19 (Oxford–AstraZeneca) and Ad26.COV2.S (Janssen), as well as RNA-based immunizations, including BNT162b2 (Pfizer–BioNTech) and mRNA-1273 (Moderna) (5). All of these vaccines have been approved due to their excellent safety and efficacy data from clinical trials (6, 7). Nevertheless, the clinical trials were underpowered to identify rare adverse events crucial for continuous risk-benefit assessments (8). The observed heightened susceptibility to cerebral venous sinus thrombosis following the ChAdOx1nCoV-19 vaccine serves as an example of a rare adverse neurological event with an immune-mediated pathogenesis (9).

The occurrence of immune-mediated neurological complications after vaccination is biologically plausible considering that vaccines can cause a strong expression of proinflammatory cytokines and a T cell response, leading to neuroinflammation after microglia activation (10). This was confirmed for peripheral complications such as GBS in the setting of adenovirus-vectored vaccines as the observed-to-expected ratios for GBS following Ad26.COV2.S vaccination were ≥1.5-fold higher than background rates in one study (11), whereas another study estimated 38 excess cases of GBS per 10 million people receiving ChAdOx1nCoV-19 (8). In both scenarios it was observed that this risk was mitigated by the fact that the rate of neurological events after acute respiratory syndrome coronavirus 2 (SARS-CoV-2) infection was up to 617-fold higher than after COVID-19 vaccination (11) and, specifically, 145 excess GBS cases per 10 million people are estimated after a positive SARS-CoV-2 test result (8). The impact of COVID-19 infection as compared to vaccination could be even more prominent, considering that a subtle neuronal damage has been demonstrated in patients with minor neurological involvement, or no symptoms at all (12, 13). In fact, neuroaxonal injury is often subclinical, and surrogate markers such as neurofilament light chain protein (NfL) are key to its detection; though significantly correlating with clinical severity, NfL also appears to be elevated in COVID-19 cases without prominent CNS manifestations (14).

Besides epidemiological evidence, a causal association can be demonstrated also if neurological complications show a specific clinical phenotype divergent from that of the naturally occurring disease. In this regard, pharmacovigilance data demonstrated that GBS occurring after administration of adenovirus-vectored vaccines present with an abnormally high incidence of facial palsy (facial diplegia in most cases), which supports a causal relationship between such exposure and this syndrome (15).

Conversely, little has been done to gather information on central nervous system (CNS) adverse events of COVID-19 vaccination for which only isolated case reports and small case series exist (16–21), despite the fact that detailed assessments of potential neurological adverse events associated with COVID-19 vaccines as well as infection are urgently needed.

Herein, we aimed at characterizing the clinical spectrum and immunological features of CNS immune-related events following SARS-CoV-2 vaccination and assessing the potential for a causal association using validated diagnostic criteria.

## 2 Methods

### 2.1 Study design and patient cohort

The present study is a multicentric retrospective cohort study of patients who developed CNS complications after SARS-CoV-2 vaccine administration between December 1st, 2020 and April 30th, 2022. Patients were included from six Italian hospitals, mostly tertiary referral centers, each covering a population in the range of 300,000-1,000,000 people (*Azienda Sanitaria Universitaria Friuli Centrale*, Udine; *Istituto di Ricovero e Cura a Carattere Scientifico Mondino*, Pavia; *Azienda Ospedale-Università di Padova*, Padova; *Azienda Socio Sanitaria Territoriale Spedali Civili*, Brescia; *Azienda Unità Sanitaria Locale Socio Sanitaria 8 Berica*, Vicenza; *Azienda Sanitaria Locale di Pescara*, Pescara) and the French National Reference Center for Autoimmune Encephalitis (AE) and Paraneoplastic Neurological Syndromes (PNS) (*Centre de Référence des Syndromes Neurologiques Paranéoplasiques et Encéphalites Auto-immunes*, Lyon, France), which provides countrywide antibody (Ab) testing and clinical care for suspected cases of autoimmune neurologic syndromes (total population covered of over 68 million people).

### 2.2 Inclusion and exclusion criteria

The inclusion criteria were as follows:

- *de novo* CNS disorders developing after SARS-CoV-2 vaccination scoring the highest level (i.e. probable) in the 2021 Butler criteria for labelling causality in neurological adverse events following immunization that is (i) onset in a typical time frame (<6 weeks from vaccination – from any dose administered); (ii) no indication of an alternative etiology; and (iii) no risk factors (5).
- Evidence for an immune-mediated etiology, i.e. patients fulfilling (i) 2016 Graus criteria for AE, acute disseminated encephalomyelitis (ADEM) or Bickerstaff’s brainstem encephalitis (22); (ii) 2015 Wingerchuk criteria for neuromyelitis optica spectrum disorder (NMOSD) (23); (iii) clinical criteria for myelitis, that is the presence of symptoms/signs of neurological dysfunction in motor and sensory tracts of the spinal cord of inflammatory etiology (24)).
- Exclusion criteria were as follows: (i) insufficient medical information to assess causality or immune-mediated etiology; (ii) recent (<6 weeks) or concomitant positive SARS-CoV-2 testing, (iii) evidence of infection of the CNS; (iv) new-onset multiple sclerosis; (v) pre-existing chronic CNS autoimmune disorders.

### 2.3 Data collection and definitions

Retrospective data were collected from medical records by the participating centers. Collected information included demographic data, date of vaccination, vaccine type and dose number, onset and type of neurological symptoms, cerebrospinal fluid (CSF) characteristics, neuroglial Abs assessed and Ab results, brain/spinal cord magnetic resonance imaging (MRI) and electroencephalography (EEG) features, final diagnosis, treatment strategies adopted, and outcome. Disability was evaluated using the modified Rankin Scale (mRS) at disease onset, clinical nadir (“worst mRS”) and last available follow up.

Neurological symptoms were classified *a priori* in the following categories: working memory deficit, altered mental status (i.e., decreased or altered level of consciousness, lethargy, or personality change), psychiatric symptoms, new focal CNS deficit, language disturbance, seizures, cranial nerve (CN) palsy, ataxia, myelopathy, movement disorder, autonomic dysfunction or central hypoventilation, and ophthalmoplegia.

Lumbar puncture data included white cell count, protein level, glucose content, oligoclonal bands (OCB), and CSF/serum albumin quotient (QAlb) assessment. A concentration >500 mg/L was considered the pathologic threshold for protein content, while a cell count ≥ 5 per µL was considered pathological for cells in the CSF (22). Albumin quotient was calculated according to the following formula: QAlb=[Alb]CSF/[Alb]serum×1000; normal QAlb values were considered as <6.5 for patients aged 15–40 years, <8.0 for patients aged 41–60 years and <9.0 for patients >60 years (25). Neuronal Abs, including those targeting neuronal cell surface (NMDAR, GABA_B_R, AMPAR, LGI1, CASPR2), intracellular (Hu, CV2/CRMP5, Ri, Ma2, Amphiphyisin, GAD65), or glial surface antigens (MOG, AQP4) were assessed when clinically indicated by the referring physicians, according to the neurologic presentation and indirect immunofluorescence (IFI) pattern observed on rat brain sections.

Neuronal Ab testing required laboratory assessment using two distinct techniques, including rodent brain tissue IFI accompanied by confirmatory testing using immunoblot with recombinant proteins (for Abs directed to intracellular antigens) or cell-based assays (for Abs against cell surface or synaptic proteins), as per current diagnostic criteria and internal diagnostic flowchart (26).

A large panel of cytokines (CXCL10, IFN-γ IL-10, IL-1β, sIL-2Rα IL-6, IL-8, TNFα) was analyzed on available CSF samples using customized ultrasensitive multiplex immunoenzymatic assays (Ella instrument, Bio-Techne, USA). All samples were taken during the acute stage (first admission to the hospital, before immunotherapy administration) and tested at the Department of Laboratory Medicine of the Udine University Hospital.

Neuroimaging studies were reviewed to assess the presence of inflammatory alterations in the brain and/or spinal cord. On available MRIs, the site predominantly involved by the inflammatory process was classified *a priori* in the following categories: limbic, white matter, cortico-subcortical, and deep gray matter. EEG alterations were analyzed in terms of location (focal vs. diffuse) and type (slowing, epileptic, extreme delta brush, or status epilepticus).

The diagnostic classifications for inflammatory disorders, which were categorized as possible, probable, or definite based on internationally accepted diagnostic criteria (22, 23), encompassed AE, Bickerstaff encephalitis, ADEM, brainstem encephalitis, myelitis, and NMOSD.

Immunomodulatory therapies adopted were recorded, namely steroid bolus, oral corticosteroids (CS), intravenous immunoglobulins (IVIg), plasma exchange (PE), rituximab (RTX), and cyclophosphamide (CP). The use of antiseizure medications (ASMs) was also collected.

### 2.4 Statistical analysis

Descriptive analysis is presented as frequencies and percentages for categorical variables and as median and range for continuous variables. Statistical analyses were performed using IBM SPSS Statistics Software V.25.0.

### 2.5 Ethical approval

The study was approved by the Institutional Review Board of the University of Udine (IRB DAME) with the following protocol number: IRB: 83/2022.

## 3 Results

Nineteen patients were included in the study (11 patients from the 6 Italian participating centers and 8 patients from the French Reference Center). The median age was 56 years (range: 20-83 years); 10/19 patients were male (53%). The most frequently administered vaccine was Pfizer-BioNTech BNT162b2 (10/19, 53%), followed by AstraZeneca ChAdOx1 nCov-19 (7/19, 37%) and Moderna mRNA-1273 (2/19, 11%). None of the patients received Janssen/Johnson&Johnson Ad26.COV2.S. Overall, 12/19 (63%) patients received an mRNA-based vaccine, while the remaining an adenovirus-vectored vaccine (7/19, 37%).

For comparison, the proportion of people receiving the different types of COVID-19 vaccine in the Italian and French population was as follows: 92% mRNA-based vs. 8% adenovirus vectored-base in Italy (from *Rapporto Vaccini Anti-COVID-19, Ministero della Salute*, accessed on 15 December 2022) and 94% mRNA-based vs. 6% adenovirus vectored-base in France (from *Agence nationale de sécurité du médicament et des produits de santé*, ANSM, accessed on 15 December 2022), suggesting a possible over-representation of the adenovirus-vectored vaccines group in the present cohort.

Neurological symptoms presented after the first vaccine dose in in 8/19 (42%), the second in 5/19 (26%), the third in 4/19 (21%), and the fourth in 2/19 (11%). The median time lag between vaccination and symptoms onset was 14 days (range: 2-41 days). A history of systemic autoimmune disease was identified in three patients (3/19, 16%), and it consisted in autoimmune thyroid disorder in all of them.

AE was the most frequent diagnosis (11/19, 58%), followed by myelitis (4/19, 21%), ADEM (3/19, 16%), and brainstem encephalitis (1/19, 5%). The level of diagnostic certainty was “definite” for most patients (10/19, 53%), and “possible” for the remaining ones (9/19, 47%).

The most frequent symptoms were altered mental status (7/19, 37%) and myelopathy (7/19, 37%), followed by subacute working memory deficit (6/19, 32%). Other symptoms included: movement disorder (5/19, 26%), seizures (5/19, 26%), new focal CNS deficit (5/19, 26%), ataxia (4/19, 21%), CN palsies (2/19, 11%), language disturbance (2/19, 11%) and psychiatric symptoms (2/19, 11%). None of the patients developed autonomic dysfunction/central hypoventilation nor ophthalmoplegia. **Table 1** summarizes the main clinical features of the patients.

**Table 1:** Characteristics of patients with CNS immune-related disorders after Sars-Cov-2 vaccination.

Patient	Sex, age (years)	Type of vaccine, dose number	Time lag vaccination-symptoms onset (days)	CNS immune-related disorder	CSF (WBCs per µL/Protein (mg/L)/OCBs/QAlb)	MRI involvement	Ab positivity	EEG	Immunotherapy	mRS at onset/clinical nadir/at last follow-up (length of follow-up, days)
1	M, 65	Oxford-AstraZeneca, 1	8	AE	n (3.8)/↑ (518)/NA/NA	n	–	Diffuse slowing	–	5/5/1 (130)
2	F, 56	Pfizer–BioNTech, 1	15	ADEM	n (4.8)/n (301)/+/NA	Bilateral white matter	–	Left temporal slow activity	Oral corticosteroids	1/2/0 (52)
3	F, 73	Oxford-AstraZeneca, 2	14	AE	n (3)/↑ (930)/+/17.1	n	–	Left frontotemporal epileptic activity	IV corticosteroids, IVIg	1/5/1 (43)
4	F, 35	Moderna, 3	19	Myelitis	n (1)/↑ (780)/-/11.0	C5-T5, longitudinally extensive	–	NA	IV and oral corticosteroids	2/4/2 (46)
5	M, 47	Pfizer–BioNTech, 2	22	AE	↑ (44)/↑ (690)/-/8.9	n	–	Diffuse slowing	Oral corticosteroids	3/4/1 (252)
6	F, 45	Oxford-AstraZeneca, 1	4	Myelitis	↑ (12)/↑ (767)/+/8.0	T7-T8 and T10-T11	–	NA	IV and oral corticosteroids	2/2/1 (394)
7	M, 68	Pfizer–BioNTech, 3	2	AE	n (2)/↑ (522)/NA/NA	Limbic, unilateral	–	n	IVIg	2/2/2 (138)
8	M, 56	Oxford-AstraZeneca, 1	41	AE	↑ (5)/n (300)/+/NA	n	Anti-Hu	n	IV corticosteroids, IVIg	2/4/1 (332)
9	M, 20	Pfizer–BioNTech, 3	7	AE	n (4)/n (374)/NA/NA	Right hemisphere and posterior fossa pachymeningitis	–	Status epilepticus	Oral and IV corticosteroids	1/4/1 (49)
10	M, 41	Pfizer–BioNTech, 3	19	AE	n (1)/n (440)/NA/NA	Bilateral temporal cortico-subcortical	–	n	IV corticosteroids, IVIg	4/4/1 (107)
11	F, 40	Oxford-AstraZeneca, 1	7	AE	↑ (23)/↑ (720)/NA/NA	n	–	Diffuse slowing	IV and oral corticosteroids, RTX, PE	2/3/1 (382)
12	M, 64	Pfizer–BioNTech, 1	14	AE	↑ (19)/n (410)/+/n	n	–	n	IV corticosteroids, IVIg	1/3/2 (32)
13	M, 60	Oxford-AstraZeneca, 1	35	ADEM	↑ (71)/↑ (800)/+/NA	Bilateral frontal, parietal and occipital periventricular white matter	–	n	IV and oral corticosteroids, cyclophosphamide	3/3/1 (540)
14	M, 83	Pfizer–BioNTech, 4	12	Myelitis	n (4)/n (470)/+/NA	Both cerebral peduncles (spinal MRI was n)	–	n	IV corticosteroids, RTX	4/4/4 (124)
15	M, 75	Pfizer–BioNTech, 4	4	AE	↑ (5)/n (450)/+/NA	Both upper cerebellar peduncles	–	NA	IV and oral corticosteroids, IVIg	3/3/0 (184)
16	F, 44	Pfizer–BioNTech, 2	10	Myelitis	↑ (9)/n (280)/-/n	Spinal	–	NA	IV corticosteroids	3/3/2 (75)
17	F, 40	Oxford-AstraZeneca, 1	20	Brainstem encephalitis	↑ (11)/n (268)/-/n	Bilateral cranial neuropathy and encephalomyelopathy	–	n	IVIg	3/3/1 (108)
18	F, 61	Moderna, 2	23	ADEM	↑ (56)/n (144)/-/n	Bilateral deep grey matter	–	Diffuse slow activity	Oral corticosteroids, IVIg	3/4/2 (167)
19	F, 41	Pfizer–BioNTech, 2	4	AE	n (1)/n (239)/-/NA	Bilateral cortico-subcortical	–	Bitemporal, left predominant epileptic activity	–	1/3/2 (411)

Ab, antibody; ADEM, acute disseminated encephalomyelitis; AE, autoimmune encephalitis; CSF, cerebrospinal fluid; EEG, electroencephalogram; F, female; IV, intravenous; IVIg, intravenous immunoglobulins; M, male; MRI, magnetic resonance imaging; mRS, modified Rankin Scale; n, normal; NA, not available/not performed; OCB, oligoclonal band(s); PE, plasma exchange; QAlb, CSF/serum albumin ratio; RTX, rituximab; WBC, white blood cell(s).

### 3.1 CSF findings

All patients underwent lumbar puncture, which in most cases showed inflammatory alterations (14/19, 74%). More than half of the patients presented CSF pleocytosis (10/19, 53%) and 8 patients increased CSF proteins (8/19, 42%). Overall, CSF-specific OCBs were found in 8/14 patients tested (57%). Albumin quotient was available in 8/19 cases (42%), and it was found elevated in half of them (4/8, 50%), including one borderline positivity (QAlb=8 in a 45-year-old patient). Autoantibodies were detected in 1/17 patients tested (6%), which consisted of neuronal (Hu) Abs (n=1; positive in serum and CSF).

**Figure 1:**
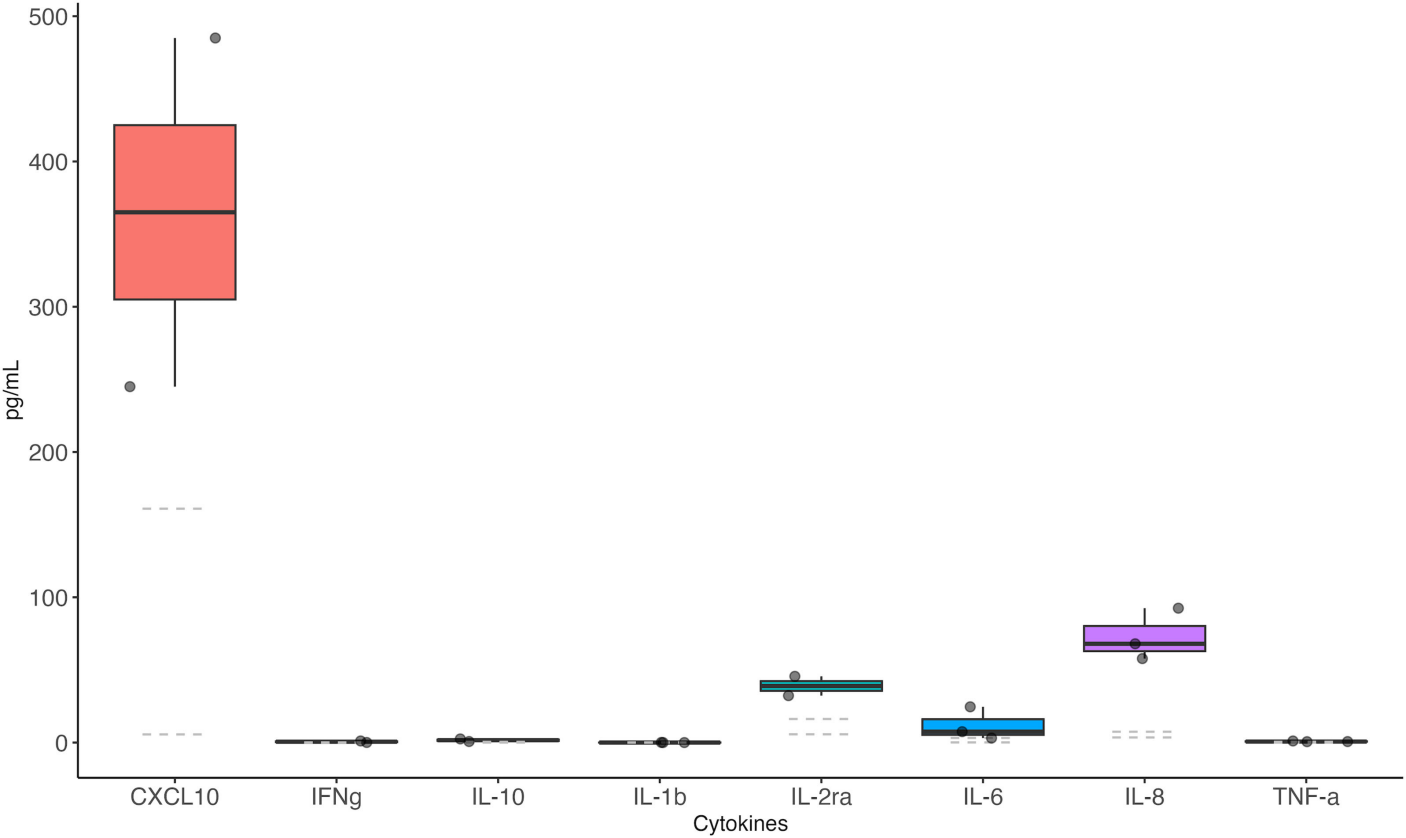
Cerebrospinal fluid cytokine levels in patients with CNS immune-mediated disorders after SARS-CoV-2 vaccination The CSF samples of 3 patients developing CNS disorders after SARS-CoV-2 vaccination were analyzed. Data refer to Case #1 (AE), Case #2 (ADEM) and Case #7 (AE). All samples were taken during the acute stage (first admission to the hospital, before immunotherapy administration). Dashed lines = reference values.

Cytokine CSF analysis was performed in 3 patients and revealed markedly increased levels of C-X-C motif chemokine ligand 10 (CXCL10) (also known as interferon gamma-induced protein 10, IP-10) in all tested cases, along with increased levels of sIL-2Ra and IL-8 (**Figure 1**).

### 3.2 Neuroimaging findings

**Figure 2:**
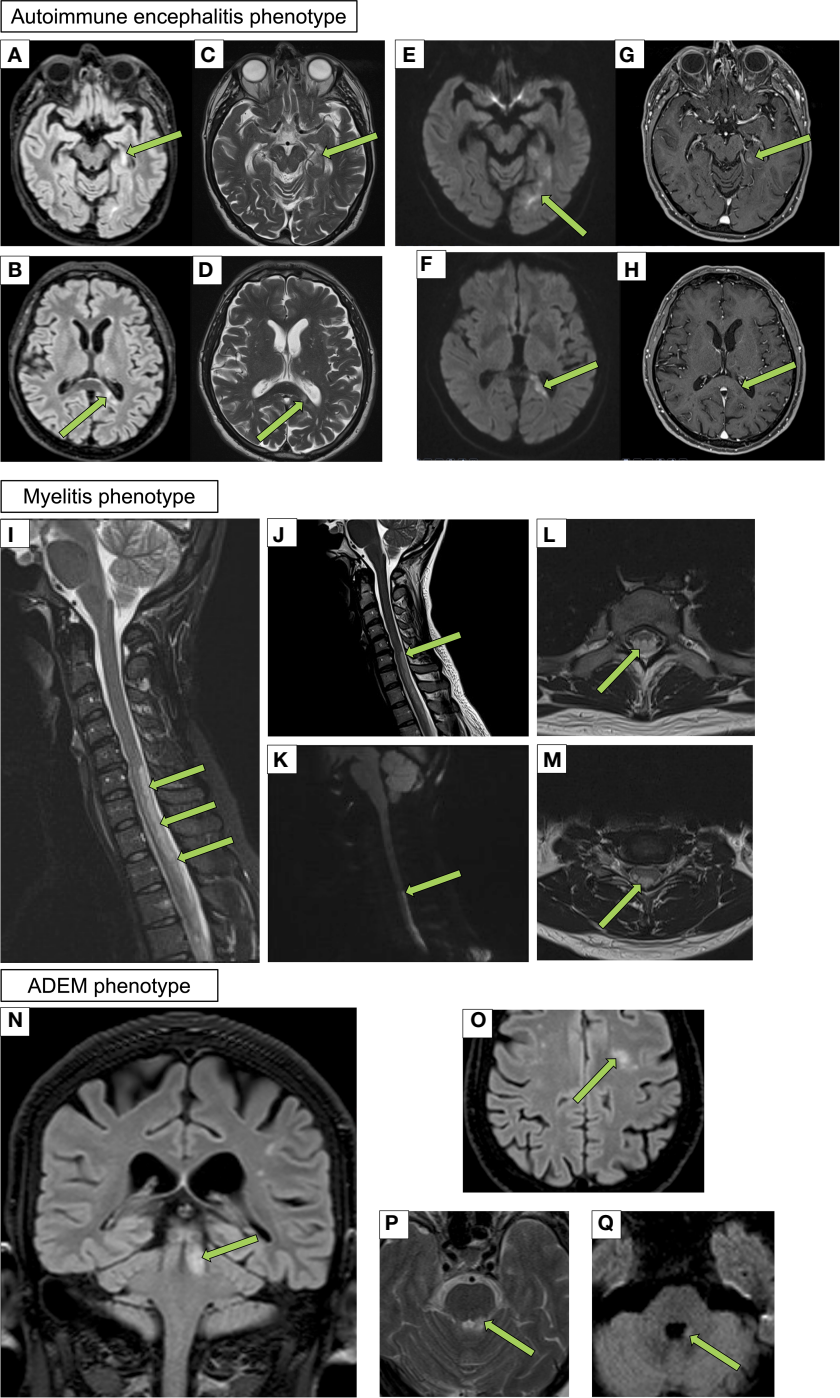
Neuroimaging findings in patients with CNS immune-mediated disorders after SARS-CoV-2 vaccination Autoimmune encephalitis phenotype (Case #7): axial fluid-attenuated inversion recovery (FLAIR) **(A, B)**, turbo spin echo (TSE) T2 weighted **(C, D)**, diffusion weighted **(E, F)** and Vibe T1 weighted after gadolinium administration **(G, H)** showing two different continuous lesions with restricted diffusion and patchy enhancement involving the left hippocampus and splenium of the corpus callosum. Myelitis phenotype (Case #4): sagittal short tau inversion recovery (STIR) **(I)**, TSE T2 weighted **(J)**, diffusion weighted **(K)** sequences and axial TSE T2 weighted **(L, M)** sequences of the cervical and upper thoracic spine showing a hyperintense cord lesion that extends over four contiguous vertebral segments. The lesion is characterized by restricted diffusion and involves the dorsal columns of the spinal cord. Acute disseminated encephalomyelitis (ADEM) phenotype (Case #2): coronal **(N)** and axial FLAIR **(O)**, axial TSE T2 weighted **(P)** and diffusion weighted imaging **(Q)** sequences showing two different hyperintense lesions also with restricted diffusion **(Q)** involving the left superior cerebellar peduncle and white matter of centrum semiovale.

All patients underwent brain and/or spinal cord MRI that showed inflammatory alterations in most of them (13/19, 68%), including 11/13 (85%) with bilateral alterations and 2/13 (15%) with unilateral alterations (**Figure 2**).

MRI findings included predominant white matter involvement (n=4), cortical and subcortical alterations (n=2), longitudinally extensive spinal cord involvement (n=2), limbic involvement (n=1), deep grey matter involvement (n=1), hemispheric and posterior fossa pachymeningitis (n=1), cranial nerve involvement and encephalomyelitis (n=1), and T7-T8 and T10-T11 T2 hyperintensity (n=1). Five patient showed contrast-enhancement (5/19, 26%). Among all brain MRI studies examined, the cerebellar peduncles were affected in 2 patients and showed contrast enhancement in all of them (2 with additional prominent white matter alterations).

### 3.3 EEG findings

**Figure 3:**
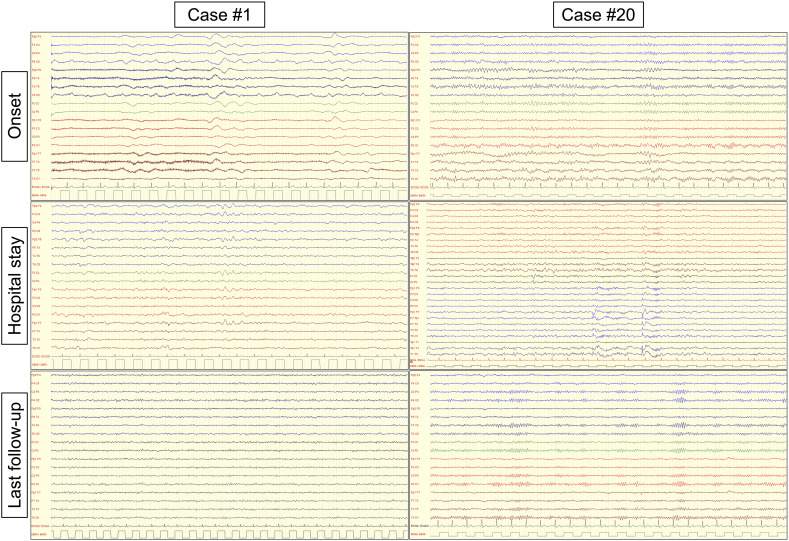
Electroencephalography findings in patients with CNS immune-mediated disorders after SARS-CoV-2 vaccination Electroencephalography (EEG) findings in two representative patients with autoimmune encephalitis ensuing after SARS-CoV-2 vaccination. Case #1: intermittent diffuse slowing (delta activity) was present at onset. During hospital stay, EEG slowing persisted mostly on the anterior (frontal) regions. At last follow-up, the patient had a normal EEG examination and clinically recovered. Case #19: mild slowing (theta activity) was present at onset, mostly over the left temporal region. During hospital stay, epileptic discharges (spike and wave complexes) were present over the left temporal region and the patient experienced focal (temporal) seizures. At last follow-up, the patient had a normal EEG examination but was still treated with antiseizure medications due to the persistence of focal seizures.

EEG was performed in all but four patients (15/19, 79%). A pathological EEG was documented in approximately half of the cases examined (8/15, 53%) (**Figure 3**). Five patients showed a diffuse EEG alteration (5/15, 33%), including diffuse slowing (n=4) and status epilepticus (n=1). Three had focal alterations (3/15, 20%), including focal slowing (n=1) and focal epileptic discharges (n=2).

### 3.4 Treatment

All patients but 2 were given immunomodulatory treatment (17/19, 89%), and many received more than 1 drug (12/19, 63%). Steroid bolus was administered to most of the patients (12/19, 63%), while oral steroids to 9 of them (9/19, 47%). Other immunosuppressive therapies were IVIg (8/19, 42%), RTX (2/19, 11%), CP (1/19, 5%), and PE (1/19, 5%). ASMs were administered to 7 patients (7/19, 37%).

### 3.5 Outcome

Disability at clinical nadir was severe (mRS=5) in 2/19 (11%) patients, moderately severe (mRS=4) in 7/19 (37%), moderate (mRS=3) in 7/19 (37%) and mild (mRS=2) in 3/19 (16%).

At the last available follow-up (median 130 days; range: 32-540), 2/19 (11%) were asymptomatic (mRS=0), 10/19 (53%) had no significant disability (mRS=1), 6/19 (32%) mild disability (mRS=2), and 1/19 (5%) moderately severe disability (mRS=4).

Importantly, the only patient positive for neuronal (Hu) Abs had a dramatic response to immunotherapy (steroid bolus and IVIg which lead to a significant mRS change 4→1) which was maintained after almost 1 year of follow-up. The patient had a persistently negative cancer screening (using both whole-body computed tomography (CT) and positron emission tomography (PET) scans and had never received immune checkpoint inhibitors (ICIs).

## 4 Discussion

In this study, we reported 19 cases of CNS immune-mediated disorders, all occurring within 6 weeks of SARS-CoV-2 vaccination, which were identified from 7 tertiary referral hospitals across Italy and France (one of them being a national referral center for AE), over almost 1 year and half of vaccination campaign. Even though this study was not designed to capture the impact of COVID-19 vaccination on the epidemiology of CNS inflammatory disorders, these data suggest that such adverse events are very rare, in line with previous reports (8, 11, 19). Conversely, the occurrence of both peripheral (e.g. GBS (27–29)) and central (e.g. AE, myelitis, ADEM (18, 30)) immune-mediated complications of COVID-19 was much higher (11), especially in the first waves of the pandemic, likely reflecting a protective role of vaccines not only in reducing SARS-CoV-2 infections, hospitalizations, and deaths but also neuroimmune disorders triggered by the infection itself (8).

Herein, we observed that three main clinical patterns (AE, myelitis, and ADEM) characterize CNS immune-mediated disorders in patients recently vaccinated from SARS-CoV-2, a type of involvement that parallels the one triggered by COVID-19 (30). Also, similarly to neuroimmune disorders induced by the infection, most of the cases were Ab-negative and cytokine analysis in a small subgroup of cases was in favor of a robust T-cell activation, with elevated levels of CXCL10 (IP-10). CXCL10 is a proinflammatory chemokine which has a key role in the priming phase of the T cell response, as well as in the recruitment of CD8^+^ and Th1-type CD4^+^ effector T cells (31). However, the small sample size prevents us from drawing any robust conclusion, and our findings require confirmation in a larger cohort of patients.

Vaccinations, as it was demonstrated for the ChAdOx1nCoV-19 vaccine (32), can lead to the production of proinflammatory cytokines and T cell responses. This can result in the release of pyrogenic cytokines into the bloodstream, similar to the response seen in natural infections (16). Interestingly, a protective clinical effect from SARS-CoV-2 vaccination is seen within 11 days, when neutralizing Abs are hardly detectable, suggesting that vaccine-induced CD8+ T cells may therefore be the main mediators of protection at this early stage (31, 33, 34). In rare cases, vaccination might lead to neuroinflammation due to microglial activation, influenced by individual genetic and immune memory factors (35). The mechanisms by which vaccinations may potentially induce *de novo* CNS inflammatory disorders include molecular mimicry, epitope spreading, or demasking/release of epitopes (17, 36). It is possible that similarities between foreign epitopes (provided by vaccine exposure) and self-antigen leads to aberrant activation of autoreactive T or B cell clones (molecular mimicry) (37). Alternatively, an initial antigenic stimulus may induce spreading of the specificity of the immune response, which includes self-epitopes other than the one which initiated inflammation (epitope-spreading) (38). SARS-CoV-2 vaccine may as well have the potential to activate autoreactive clones in an antigen-independent, non-specific manner, mediated by indirect signals that favor an inflammatory milieu (bystander activation) (39). It is difficult to hypothesize which of these mechanisms could be involved, and whether different classes of autoimmune complications may underlie different pathogeneses. Interestingly, a recent paper on acute inflammatory diseases of the CNS after SARS-CoV-2 vaccination has shown that almost all patients included with detectable autoantibodies received ChAdOx1S, suggesting that different types of vaccines may trigger specific forms of CNS autoimmunity (17). In addition, vaccination might also trigger the exacerbation of pre-existing, subclinical, neuroimmune disorders (36), similarly to what it was observed for other adverse events triggered by nonspecific T cell activation, such as those of ICIs (40, 41). Finally, a temporal coincidence of the 2 events (vaccination and CNS immune-mediated disorders) cannot be completely excluded considering the high global vaccination rates (18, 36).

In this cohort, the diagnosis was supported by:

- the typical time frame (median of 14 days after vaccination) similar to what it was reported by Zuhorn et al. (7-11 days) (16), Abdelhady et al. (9.97 days) (42), Jarius et al. (13 days) (36), Asioli et al. (13 days) (20), Maramattom et al. (5-20 days) (19), which is a biologically plausible time frame for a CNS immune-related adverse event, considering that at that time a fully functional spike-specific CD8+ T cell response is already elicited (31, 33, 34);
- the occurrence after the first or, less frequently, the second dose in most of the patients, as previously described (17, 19, 42) and, again, in line with the mechanistic hypothesis of these disorders;
- the higher-than-expected rate of patients receiving adenovirus-vectored vaccines observed here, even though vastly more patients have been immunized with mRNA vaccines in Italy, France, and worldwide. This finding was confirmed by many studies exploring neurological complications of SARS-CoV-2 vaccination by looking at epidemiological data (8, 11), as well as by clinical studies focused on GBS (15), MOGAD (17, 36), and AE (16, 42), thus suggesting a potential role of the adenoviral vector. In addition, the magnitude of spike-specific T cell induction was demonstrated to be higher in adenovirus-vectored vaccines as compared to those mRNA-based (43);
- the characteristic symptoms previously described in the Vaccine Adverse Event Reporting System (VAERS) as rare but possible vaccine reaction of the COVID-19 vaccine (11), and previously reported also for other types of immunization (1);
- the adoption in this study of stringent methodological approaches to assigning an immune-mediated etiology as well as a probable causal association between the two events (5);
- the favorable response to immunosuppressive therapy with corticosteroids obtained in most cases.

Interestingly, all but 1 patient with an AE phenotype were neuronal Ab-negative, while the remaining patient was positive for Hu Abs (confirmed using 2 distinct techniques at the French Center for PNS). This case showed several distinctive features as compared to the classic anti-Hu phenotype, which belongs to the “high-risk” Ab group, being associated to cancer (usually small-cell lung cancer) in >85% of cases (26). Few non-paraneoplastic cases have been described in children (44), but none after vaccination. In addition, the response to treatment is usually scarce and unsatisfactory (45). Conversely, a prolonged beneficial response to first-line treatments only (corticosteroids and IVIg) was observed in this case, while multiple oncological screenings (including whole-body PET) were negative. Although we need to carefully follow this case over time before making a conclusion on the possible causal link with vaccination, it is interesting to note that an increase in the detection of Hu-Abs (along with GFAP-Abs) was observed at the Barcelona Referral Center by comparing the pre–COVID-19 (2017–2019) and COVID-19 (2020–2021) periods, while the authors did not detect a substantial increase of encephalitis mediated by Abs against neural-surface antigens (46). Supporting this observation, most (>90%) of the cases of AE temporally related to vaccination are Ab-negative in both the present series and previous systematic review of the topic (42). Few cases of anti-LGI1 AE developing after COVID-19 vaccination have been reported, including 4 cases from Italy (20) and 1 from Israel (21), but none of the AE patients in the present series tested positive for LGI1-Abs and none showed the characteristic faciobrachial dystonic seizures pathognomonic of the disorder.

LGI1-Ab AE, being an immunoglobulin G4 (IgG4)-Ab disorder, is strongly associated with particular human leukocyte antigen (HLA) class II haplotypes, in particular DRB1*07:01, which is carried by nearly 90% of anti-LGI1 patients (47). Interestingly, an association between the HLA-DRB1*07:01 allele and greater immunogenicity after the administration of mRNA-1273 vaccine was observed (48), suggesting that future studies aiming at better exploring the immunogenetic factors associated with the development of these rare adverse events are definitely warranted.

Another interesting observation from the present study was the neuroradiological involvement of cerebellar peduncles in 2 cases, all showing contrast enhancement, which was also suggested in previous reports (17, 19, 42, 49).

This study is limited by its retrospective nature and the small sample size. Also, the multicenter design does not allow us to comprehensively study the potential effect of both COVID-19 infection and vaccination on the epidemiology of these conditions.

However, this represents one of the largest series of CNS disorders developing in close temporal association with vaccination, in which a probable link with immunization was established using validated diagnostic criteria.

In conclusion, CNS immune-mediated adverse events of COVID-19 vaccination appear to be very rare and consist mostly of Ab-negative AE, myelitis, and ADEM developing approximately 2 weeks after vaccination, when a mounting robust CD8+ T cell response is suggested by the increased CXCL10 CSF levels. Clinicians should be aware of these rare complications, as most patients improved following immunomodulatory treatment. Conversely, neuroimmune complications after COVID-19 infection are far more frequent, therefore neurologists should continue to promote vaccination as benefits largely outweigh risks at the population level.

## Data availability statement

The original contributions presented in the study are included in the article/supplementary material. Further inquiries can be directed to the corresponding author.

## Ethics statement

The studies involving humans were approved by Institutional Review Board (IRB-DAME). The studies were conducted in accordance with the local legislation and institutional requirements. Written informed consent for participation was not required from the participants or the participants’ legal guardians/next of kin because the study was retrospective, observational, and non-interventional, focusing solely on the analysis of pre-existing records, without direct participant interaction or intervention.

## Author contributions

AV: Conceptualization, Data curation, Funding acquisition, Methodology, Supervision, Validation, Writing – original draft, Writing – review & editing. ST: Data curation, Formal Analysis, Investigation, Writing – review & editing. MD: Data curation, Formal Analysis, Investigation, Writing – review & editing. MF: Investigation, Methodology, Writing – review & editing. FB: Formal Analysis, Software, Writing – review & editing. MB: Investigation, Writing – review & editing. LV: Investigation, Writing – review & editing. DB: Investigation, Writing – review & editing. MG: Investigation, Writing – review & editing. PT: Investigation, Writing – review & editing. MZ: Investigation, Writing – review & editing. LZ: Investigation, Writing – review & editing. APi: Investigation, Writing – review & editing. APa: Investigation, Writing – review & editing. MV-G: Investigation, Writing – review & editing. VD: Investigation, Writing – review & editing. GG: Resources, Writing – review & editing. JH: Investigation, Writing – review & editing. MV: Funding acquisition, Resources, Writing – review & editing.
